# Retraction: Detection and Functional Characterization of a 215 Amino Acid N-Terminal Extension in the *Xanthomonas* Type III Effector XopD

**DOI:** 10.1371/journal.pone.0190773

**Published:** 2018-01-02

**Authors:** Joanne Canonne, Daniel Marino, Laurent D. Noël, Ignacio Arechaga, Carole Pichereaux, Michel Rossignol, Dominique Roby, Susana Rivas

The authors and editors retract this publication [1] following concerns raised around the images presented in several figures.

After the publication of this article, concerns were raised to the journal that upon adjustment of brightness, contrast, and/or other image settings, several inconsistencies and lines appear in Figures 2, 4, 5, and 6.

- In Figure 2, inconsistencies and lines in the HA blot image suggest this panel may have been generated by splicing multiple image fragments.
- In Figure 4B, the 6His-XopD^1-760^ blot appears to include an additional image overlaying the original blot in the lane for sample #26.
- In Figure 4B, the 6His-XopD^216-760^ blot appears to include several unmarked areas of splicing.
- In Figure 5B, there appears to be a potential splice line between the first and second lanes of the GFP blot.
- In Figure 5E, in the GFP blot, differences in background patterns/coloring suggest that the image may have been altered in the area above the bands in lanes 3 and 4.
- Figure 6D appears to include some unmarked splicing in both HA blots, between lanes 3 and 4 for each.
- In Figure 6D, lanes 3 and 4 for the Total Extract GroEL blot appear to be the same.

In addition, it was raised that the Ponceau S stained control gel shown in Figure 6B is highly similar to a Ponceau S panel shown in Figure 2B of another article by this research group [2].

The authors indicated that images were assembled inappropriately during preparation of Figures 4B, 5B, and 5E. They also confirmed that Figure 6B involves a duplication of the Ponceau S-stained control band from their previous article [2]. The authors did not provide comments regarding Figures 2 or 6D, and they noted that original blots and gels for the above-mentioned figures are unavailable.

In light of these concerns, the authors and editors retract this article. The authors apologize to the scientific community for the errors published in this article.

The journal editors notified representatives at INRA and CNRS of this matter, and contacted all co-authors about this retraction. SR made the initial request for retraction; SR, DR, DM, LN, MR agreed with the retraction; JC, IA, CP did not respond.
